# A Short Overview on Graphene and Graphene-Related Materials for Electrochemical Gas Sensing

**DOI:** 10.3390/ma17020303

**Published:** 2024-01-07

**Authors:** Mallikarjun Madagalam, Mattia Bartoli, Alberto Tagliaferro

**Affiliations:** 1 Department of Applied Science and Technology, Politecnico di Torino, Duca degli Abruzzi 24, 10129 Turin, Italy; mallikarjun.madagalam@polito.it; 2 National Interuniversity Consortium of Materials Science and Technology (INSTM), Via Giuseppe Giusti, 9, 50121 Florence, Italy; 3 Center for Sustainable Future Technologies (CSFT), Istituto Italiano di Tecnologia (IIT), Via Livorno 60, 10144 Turin, Italy; 4 Faculty of Science, OntarioTech University, Simcoe Street North, Oshawa, ON L1G 0C5, Canada

**Keywords:** graphene derivatives, electrochemical sensing, graphene tailoring

## Abstract

The development of new and high-performing electrode materials for sensing applications is one of the most intriguing and challenging research fields. There are several ways to approach this matter, but the use of nanostructured surfaces is among the most promising and highest performing. Graphene and graphene-related materials have contributed to spreading nanoscience across several fields in which the combination of morphological and electronic properties exploit their outstanding electrochemical properties. In this review, we discuss the use of graphene and graphene-like materials to produce gas sensors, highlighting the most relevant and new advancements in the field, with a particular focus on the interaction between the gases and the materials.

## 1. Introduction

The production of highly sensitive materials for electrochemical sensing is a matter of great relevance for analytic science. Actually, research is focusing on finding the best trade-off between the performance and the toughness of electrode materials [1]. In this field, graphene and graphene-related materials (GRMs) can play a game-changing role.

The outstanding electrical properties of graphene combined with its superior mechanical and optical properties have attracted great interest in electrochemical sensing applications due to the achievable sensitivity, rapid response times, and versatility in detecting a wide range of analytes never reached before [2]. The integration of graphene into electrochemical sensors has led to significant performance improvements in several fields of application, including environmental science [3], medical diagnostics [4] and quality control [5]. Graphene’s astonishing performance is due to the improvement of electron transfer at the electrode interface by a combination of electronic and chemical features [6]. Furthermore, graphene and GRMs’ high mechanical strength and flexibility further contribute to their utility in electrochemical sensing, ensuring the stability and longevity of sensors even under challenging conditions [7].

The tunability of graphene and GRMs allows their use across several types of sensors, including amperometric, potentiometric, and impedimetric ones. In amperometric sensing, the current generated by the electrochemical reaction at the electrode surface is measured and correlated with the concentration of the analyte. Graphene and GRMs contribute to sensitivity increments due to the combination of electrical conductivity and large surface area [8,9]. Potentiometric sensors measure the potential difference between a reference electrode and a working electrode, and the incorporation of graphene and GRMs enhance the stability and selectivity of the sensor, allowing them to be used in pH sensing [10] or ion detection [11]. Impedimetric sensors exploit changes in the impedance of the electrode interface upon interaction with the target analyte, and graphene’s conductivity and charge transport properties increase both the response rate and the sensitivity of impedimetric sensors [12]. Among all the possible applications, gas detection represents a critical application of GRMs in electrochemical sensing due to the great deal of attention that monitoring air quality [13] and ensuring workplace safety [14] have gained. GRM-based sensors are of particular interest due to their ability to selectively interact with specific gases that are able to induce changes in the electric signals detected [15]. This ability, together with the other outstanding properties of GRMs, is of paramount relevance for a new generation of highly sensitive tough materials for multiple gas sensing.

In this work, we report the most relevant achievements in the GRM-based electrodes field, focusing on pristine graphene, graphene oxide (GO) and reduced graphene oxide (rGO) and their tailored derivatives. We summarize the key electronic properties of graphene and GRMs and diffusely discuss their applications in gas sensing applications, focusing on CO_2_, CO, H_2_, NH_3_, NO_x_, H_2_S and SO_2_. We provide a concise and easy-to-be-exploited overview aimed to represent a reference point for researchers interested in approaching electrochemical sensing using neat and tailored graphene and GRMs in gas sensing.

## 2. Graphene and GRM Electrical Properties

**Figure 1:**
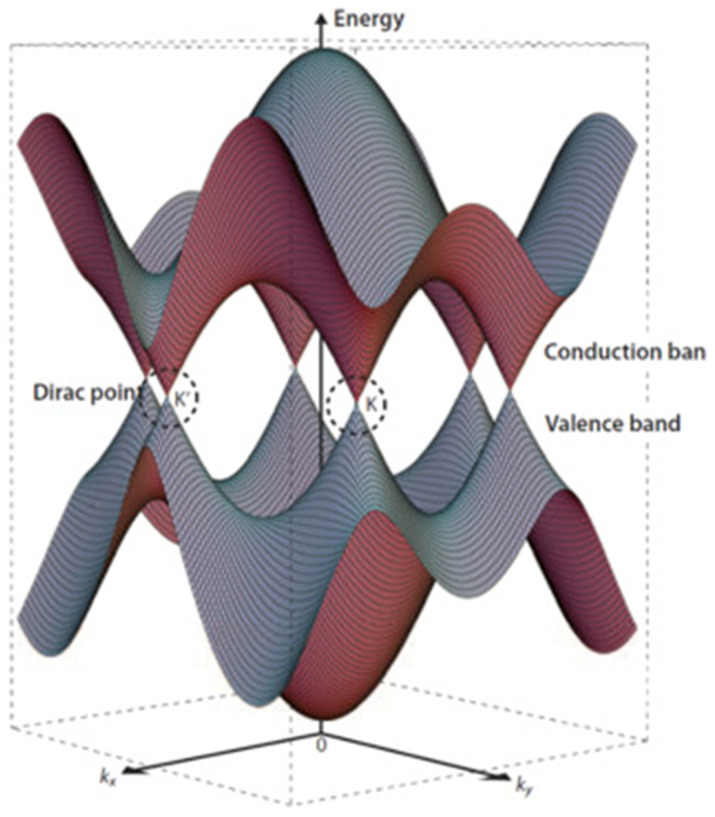
Three-dimensional schematic diagram of band structure near the Fermi level of graphene with Dirac points K and K′ highlighted. Reproduced, adapted and reprinted with permission from Lavagna et al. [21].

In agreement with the International Union for Pure and Applied Chemistry Golden Book, graphene is defined as “a single carbon layer of the graphite structure, describing its nature by analogy to a polycyclic aromatic hydrocarbon of quasi infinite size” [16]. A pristine graphene layer is composed of a planar arrangement of carbon atoms bonded through three σ bonds with the p orbitals perpendicular to the sp^2^ plane, allowing a full delocalization of the π bonds [17,18,19]. This is the reason behind graphene’s exceptional electrical properties, particularly its in-plane electron mobility. At room temperature, the electron mobility in graphene can reach up to 15,000 cm^2^ V^−1^s^−1^ [20] due to a peculiar band organization formed by two conical points in the electronic band diagram known as Dirac points, as shown in Figure 1.

The linear dispersion around Dirac points contributes to the massless nature of charge carriers in graphene, allowing them to travel at incredibly high speeds without any significant scattering due to the eventual topological disorder due to the temperature [22]. Furthermore, graphene is characterized by a relevant Hall effect, with the plateaus occurring at half integers of 4 e^2^/h rather than 4 e^2^/h. Near-ideal graphene sheets show a pronounced Hall effect, while multilayer samples show a much weaker gate dependence due to the electric field screening promoted by the other layers [23]. Interestingly, the use of a high magnetic field combined with cryogenic temperatures induces a quantum Hall effect for both holes and electrons [24,25]. The superb electrical properties of graphene are, however, counterbalanced by the absence of a band gap. A great effort has been devoted to creating graphene-like materials with a proper band gap [26], and solutions such as graphene nanoribbons have been developed [27]. Graphene nanoribbons can be shrunk by modifying the charge carrier momentum in the transverse direction, resulting in a band gap opening based on the ribbon width [28]. Alternatively, graphene can be doped with nanostructures and heteroatoms [29].

The most popular and useful procedure to dope graphene is oxidation with the formation of GO. GO is rich in oxygen functionalities such as epoxide and hydroxyl groups on its basal lattice, while carbonyl and carboxylic residues are more abundant on the edges, as described by the Lerf-Klinowski model [30]. The electronic properties of GO are strictly related to the degree of oxidation, as reported by Krishnamoorthy et al. [31]. The increment of GO oxidation induced a reduction of electron mobility [32] as a consequence of the introduction of more defects in the lattice structure of graphene. These defects act as scattering centers for charge carriers, hindering the smooth movement of electrons through the material. Nevertheless, the relationship between oxidation degree and electron mobility is a complex mix of factors, such as the oxygen functional groups and their distribution on the graphene lattice. Interestingly, GO shows a band gap due to the presence of the same defect that reduces the charge carrier’s mobility. The oxygen functionalities alter the electronic configuration of the graphene plane, disrupting the π-conjugated system and forming localized states within the energy band structure of GO, giving rise to a band gap [33]. The presence of a band gap in GO promotes a semiconducting behavior contrary to pristine graphene that shows metallic conductivity [34]. This semiconducting behavior makes graphene oxide well-suited for applications in electronic devices where a controllable on/off state is essential [35]. An interesting compromise between the conductivity of pristine graphene and the properties of GO is the reduced form of GO, named rGO. rGO is produced using harsh reductive processes [36] for decreasing the oxygen residues of GO and trying to find a balance between pristine graphene and GO with a carbon/oxygen ratio ranging from 0.4 up to 13 wt% [37]. The electrical properties of rGO are far higher than those of GO but considerably inferior to graphene, while dispersibility showed an opposite trend [38].

## 3. Graphene and Graphene-Related Materials’ Electrochemical Sensing Performance

Graphene and GRMs show several key features that allowed the spread of their use in electrochemical sensing applications. Firstly, GRMs are highly sensitive to the surrounding chemical and physical environment [39,40,41]. This is of particular interest considering the interaction with gas molecules that are adsorbed on GRMs’ surface [42] that are able to alter the electronic conductivity [43]. The conductivity alteration induced by adsorbed gas prevents the correct interaction between the dangling π-orbitals and neighboring atom orbitals, altering the conduction bands and reducing the charge carrier’s mobility. This phenomenon can be used to quantify the number of adsorbed molecules through simple electrochemical measurements in which GRMs represent the working electrode. Furthermore, the interactions between gaseous molecules and GRMs can be easily tuned by tuning the graphene functionalization, increasing both the electrochemical performance and the selectivity of the system. These features, together with a fast response and recovery of the electrodes, have boosted the use of GRMs as electrochemical gas sensing platforms, as summarized in Table 1.

GRMs showed some key advantages over other 2D materials such as MXenes, mostly focused on their preparation and tailoring. The synthesis of MXenes is a complex multistage process that should operate in well-controlled conditions [96] for the production of a high-quality material, similar to the single-layer graphene process. Nevertheless, GRM production has been developed and optimized for scalability, as proven by the production of GO and rGO from a wide range of cheap feedstocks under mild conditions through robust processes [97,98,99]. Furthermore, the carbonaceous low-dimensional materials can exploit a wide range of reactivity, fostering an easy chemical tailoring and preserving their properties [100].

### 3.1. Pristine Graphene and GRM Sensing Performance in Electrochemical Gas Detection: CO_2_ and CO

**Figure 2:**
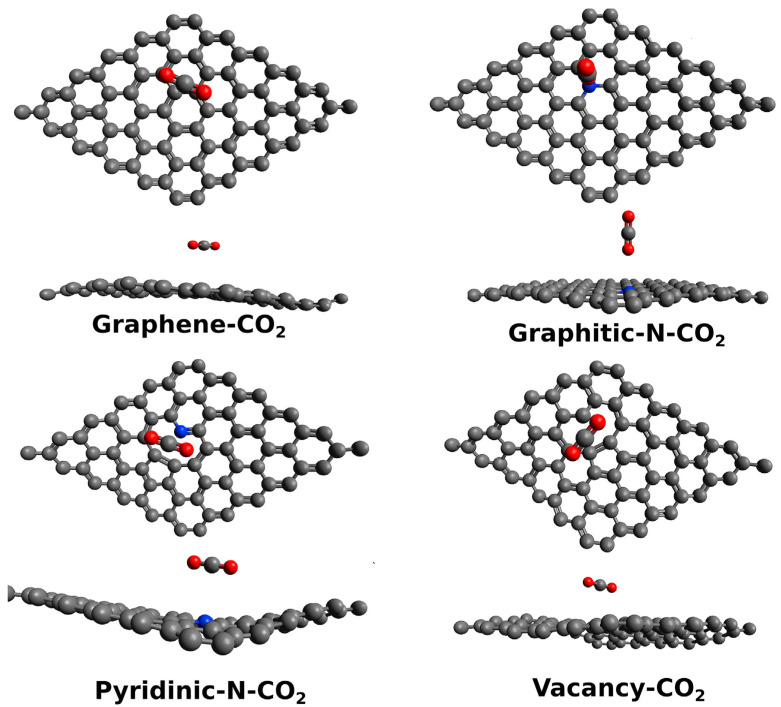
Computational simulation of the interaction between CO_2_ and graphene or GRMs at 25 °C (carbon atoms were reported as black, oxygen atoms were reported as red and nitrogen ones as blue). Reprinted with all permission from del Castillo et al. [102].

Monitoring the asphyxiating gases produced from combustion, such as CO_2_ and CO, is a relevant safety issue [101]. The main issue of detecting CO_2_ through electrochemical sensing is the interference of other species in the atmosphere, such as water, CO and oxygen. Smith et al. [44] investigated the cross-sensitivity of a capacitive CO_2_ sensor in the presence of several residual atmospheres (Ar, H_2_O, N_2_) using a chemical vapor-deposited single-layer graphene. Particularly, the authors investigated the effect of humidity on the sensor performance, showing the absence of sensitivity towards CO_2_ in the presence of atmospheric-level humidity. The authors simulated through density functional theory (DFT) calculations the effect of H_2_O and CO_2_, showing that the reduction in sensitivity towards CO_2_ was due to the electronic alteration of graphene induced by the adsorbed water molecule. The study of CO_2_ with GRMs is of great interest for producing high-performance gas sensors, and it is affected by several key factors, such as doping, as reported in Figure 2.

Castillo et al. [102] investigated the role of heteroatom-doped graphene in sensing CO_2_, proving that the presence of nitrogen graphitic sites can alter the local morphology of graphene sheets and improving the sensitivity towards CO_2_ over that achievable by using a Pt-decorated electrode. The authors suggested that this was due to the very same nature of nitrogen graphitic sites that act as p-type doping agents. This induced a pullout of electrons from CO_2_, improving the electrocatalytic activity of the nitrogen-doped graphene.

Deji et al. [103] evaluated the effect of boron and phosphorous co-doping of graphene nanoribbons for direct CO_2_ detection using first-principle DFT simulation. The authors reported that phosphorous-doped graphene showed an adsorption energy eight times higher than pristine material, while the boron-doped one outperformed it. This study is of particular significance as it assesses the relevance of the doping agent. Additionally, Elgammal et al. [104] also proved that the support onto which graphene is deposited affects the sensing process, even if not in such a relevant way. The authors modelled the performance of graphene supported on silica or sapphire substrates for detecting both CO_2_ and H_2_O molecules using DFT simulations. The results showing the differences between the substrates are in the range of 1 to 10 meV. Interestingly, authors reported that H_2_O molecules prefer to be adsorbed onto hollow sites in the center of the graphene hexagonal moieties, while CO_2_ molecules prefer sites bridging carbon–carbon bonds or directly on top of carbon atoms. Also, the authors reported that the adsorption energy of CO_2_ was up to 0.17 eV, while H_2_O showed values close to 0.09 eV.

The weak interactions between CO_2_ and graphene are a relevant issue, but several studies reported the possibility of using GRMs as solid materials for CO_2_ sensing. Yoon et al. [45] assembled a CO_2_ sensor fabricated by mechanical cleavage of nanographite plates. The authors were able to detect CO_2_ at room temperature in the presence of water (humid conditions), observing a linear response of conductance in the range between 10 and 100 ppm. Fan et al. [46] used a double-layer impedimetric electrode for the detection of CO_2_ without observing any significant influence of H_2_O for relative humidity (RH) up to 3%. The authors also proved that double-layer graphene performed better than single-layer due to the different spatial distribution of the electronic density.

GRMs have also been diffusely used for improving the interaction with CO_2_. Akhter et al. [47] designed a low-cost, low-power, miniature, highly sensitive and selective impedimetric CO_2_ sensor using GO. The authors reported a linear range from 400 ppm to 4000 ppm with good performance in reproducibility and stability. Furthermore, they also achieved a very negligible cross-sensitivity with H_2_O and a fast response and recovery rate. Muhammad Hafiz et al. [48] used rGO produced by hydrogen plasma as an impedimetric sensor. The authors reported a CO_2_ gas-sensing response of 71% (calculated as resistance variation of the electrode) in the presence of a CO_2_ concentration up to 1500 ppm in N_2_ and 37% RH, while the performance decreased down to a response of 15% in air environment with 68% RH. Nevertheless, the sensor showed a fast response and a good recovery rate. Alternatively, GRMs that include metal species can be used, as reported by Miao et al. [49]. The authors developed a platform system able to operate from 10 up to 60 °C with 97% RH and a CO_2_ linear response ranging from 300 up to 1100 ppm.

**Figure 3:**
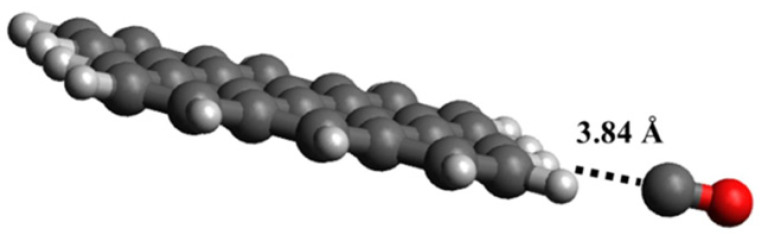
Computational simulation of the interaction between CO and graphene at 25 °C(carbon atoms were reported as black, oxygen atoms were reported as red and hydrogen ones as white). Reprinted with all permission from Dindorkar et al. [105].

CO showed a different interaction geometry with graphene, as reported by the computational study of Dindorkar et al. [105] and summarized in Figure 3.

While CO_2_ preferentially interacts with carbon atoms, the CO preferential interaction is with the edges of graphene sheets (Figure 2). The authors also found that CO interacts directly with carbon atoms but only in highly doped fragments containing silicon carbide or boron nitride domains. Similar effects were reported in the presence of GO by Deji and co-workers [106] that also proved the effectiveness of the tailoring process, with metal nanoparticles decreasing the adsorption energy up to 40 times compared with pristine graphene [107]. Metal oxides combined with graphene and GRMs are able to form p-n junctions, increasing the conductivity and improving sensing performance [108]. When it comes to CO sensing, Pd has shown the most remarkable performance, as reported by Kashyap et al. [50], using palladium-tailored rGO as an impedimetric sensor. The authors tested the response to CO in the presence of both CH_4_ and H_2_, suggesting that the interaction mechanism of CO was lying between the sole interactions with electron withdrawing or electron donating. As reported by Shojaee et al. [51], the combination of Pd with a metal oxide such as SnO_2_ could be particularly beneficial for both response and recovery, improving, at the same time, the specific surface area of the material. The authors also provided an overview of the effect of RH on a 400 ppm CO sample analysis, reporting a decrement of response for RH up to 60%. They ascribed this behavior to the competitive adsorption of CO and water molecules on the Pd and SnO_2_. A further RH increment of up to 85% induced a considerable increment of electrode response due to the formation of surface conductive channels [67]. The surface porosity was also investigated by Ha et al. [52] using ZnO nanoparticles onto rGO. The authors achieved an electrode response value of 85% for 1000 ppm CO at 200 °C, with a recovery time of 9 s. Similarly, the response value, response time, and recovery time of the sensor at room temperature were 27.5%, 14 s, and 15 s, respectively. The sensor demonstrated a distinct response to various CO concentrations in the range of 1–1000 ppm and good selectivity towards CO gas. In addition, the sensor exhibited good repeatability in multicycle and long-term stability. Neetha et al. [53] decorated rGO with Mn_3_O_4_, achieving a response time of only 3 s at 25 °C using 50 ppm of CO. Similar results were obtained using SnO_2_ on graphene [54], CuO on rGO [56] and mixed metal oxide over rGO [55,57].

Nevertheless, inorganic tailoring is not a mandatory condition for detecting CO. rGO by itself can act as an active material for the detection of CO, as reported by Panda et al. [58]. The authors achieved a 71% sensitivity using 30 ppm CO at room temperature (RT), with a recovery time of up to 30 s and a remarkable sensitivity of up to 10 ppm. The authors suggested that the performance was due to the in situ production of atomic, ionic, and radical oxygen sites, which play a relevant role in both the adsorption of CO and electronic density rearmament. Furthermore, GRMs can be functionalized with polymers, as reported by Farea and co-workers [59,60] and by Mohammed et al. [61], or by metal organic frameworks boosting the overall CO sensing performance, as reported by More et al. [62].

### 3.2. Pristine Graphene and GRM Sensing Performance in Electrochemical Gas Detection: H_2_

**Figure 4:**
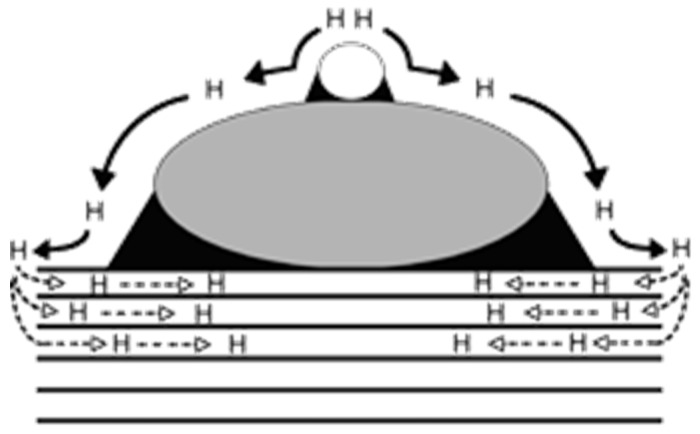
Schematic representation of primary and secondary spillover promoted by the presence of metal nanoparticles and GRMs. Reprinted with all permission from Lachawiec [109] (*Copyright © 2005, American Chemical Society*).

H_2_ is among the most elusive gases to be detected, and neat graphene cannot be used for direct sensing of it. The most common strategy for H_2_ sensing is tailoring the GRM surface with metal nanoparticles, activating a mechanism known as spillover, as reported in Figure 4.

H_2_ spillover is a complex phenomenon occurring when H_2_ molecules dissociate onto a metal particle and diffuse as atomic hydrogen to the graphene support, while the second spillover involves a further transportation towards the carbon support. This behavior can be modulated by the introduction of layered GRMs, as reported by Kumar et al. [110], who modelled H_2_ sensing in a GRM containing a layer of antimonene. DFT calculations showed the presence of a Bader charge transfer mechanism from the antimonene layer towards the graphene one that was able to change the potential barrier from the Ohmic to the Schottky type. Moving to tailored GRMs, Pd and Pt are the higher-performing metals due to their ability to interact with hydrogen through adsorption and release processes [111,112,113,114,115,116,117,118]. As reported by Kishnani et al. [119], palladium-doped or -decorated graphene is very effective in sensing H_2_. Particularly, the authors reported a higher electrochemical activity and conductivity for palladium-decorated graphene compared with the palladium-doped one, while the charge transfer and recovery time showed an opposite trend. Chung et al. [63] decorated a single-layer graphene sheet with palladium nanoparticles of 3 nm of average size. The authors reported a response of 33% using 1000 ppm of H_2_ at 25 °C and a remarkable detection limit of 20 ppm. The effect of palladium active centers was also observed by Lange et al. [120] using cyclic voltammetry without providing any highlights on the mechanism. Lee et al. [64] incorporated palladium nanoparticles into a 3D-GRM structure, reaching a response of 41.9% under 3% H_2_ residual atmosphere.

Platinum has also been investigated as a viable alternative to palladium. Lu et al. [65] decorated rGO with platinum nanoparticles by using freeze-drying-assisted techniques, reaching a sensitivity toward 0.5% hydrogen up to 8% and a recovery time of 63 s. Similarly, Lee et al. [64] and Phan and co-workers [66] produced a highly porous 3D-GRM containing platinum nanoparticles, achieving good linearity from 1 to 100 ppm. As reported by Russo et al. [67], the addition of SnO_2_ to palladium-decorated rGO was particularly beneficial, enhancing the response of palladium-decorated GRMs over four times. The authors suggested that the enhancement of sensing performance was due to the formation of a heterojunction between the n-type SnO_2_ and the p-type rGO in the heterostructure, boosting the catalytic effect of platinum in promoting the dissociation of H_2_.

Non-noble metal oxides have also been used extensively coupled with GRMs for H_2_ detection. Ahmad Fauzi et al. [68] decorated a proton-conducting GO membrane with WO_3_, producing a potentiometric H_2_ sensor. The authors claimed a response of 50 mV in the presence of H_2_ 100 ppm in air atmosphere and a detection limit of 11 ppm. ZnO was also used with GO, with interesting results, as reported by Rasch et al. [69]. The authors achieved a very low detection limit of 4 ppm, with a very fast response of around 114 s and a small recovery time of 30 s.

### 3.3. Pristinine Graphene and GRM Sensing Performance in Electrochemical Gas Detection: H_2_O

**Figure 5:**
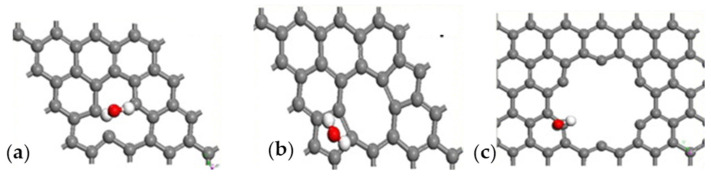
Computational simulation of the interaction between H_2_O and graphene at 25 °C in (**a**) vacancy defect, (**b**) 5–7 defect and (**c**) close to hole defect. Reprinted with all permission from del Ye et al. [122].

Humidity sensors play a critical role in several industrial sectors, such as semiconductor production, in which moisture content in the air is a critical parameter [121]. GRMs provide interesting solutions to detecting the moisture content of air, even at low concentrations, due to the interaction occurring between the graphene surface and H_2_O molecules, as sketched in Figure 5.

As reported in Figure 3, H_2_O molecules interact with graphene sheets without bonding and only through weak interactions with a distance of 2.5 Å and a higher deformation due to the interaction close to hole defects (Figure 5c), while the other cases (Figure 3a,b) did not show any significant distortion. Wang et al. [70] utilized vertically aligned graphene arrays as a humidity sensor platform. The authors observed the rise of system current with the increment of RH, suggesting a link to the Schottky barrier height with the junction resistance decrement due to the adsorption of vapor molecules. The authors suggested that the water molecules act as electron acceptors, increasing the hole density in the graphene systems. Zeng et al. [71] produced a self-powered H_2_O flexible sensor with ultrafast response and recovery time of up to 0.3 s using GO. The authors claimed to have obtained a faster response in the field of humidity sensors, and they were able to operate in an RH range from 33 to 98%. Interestingly, they proposed an interpretative model of H^+^ diffusion occurring during the sensing process based on Grotthuss hopping [123]. Yu et al. [121] produced a sensor based on rGO with the highest sensitivity for humidity. The authors suggested that this exceptional behavior was due to the spherical double surfaces and small pores in the 3D structure of rGO, allowing an optimal exposure of functionalities and a magnification of H_2_O interactions. Nevertheless, both GO and rGO suffer several issues, such as cross-reactivity towards the other gas present in the analyte. Seeneevassen et al. [124] used a GO sensor to monitor the humidity in an effluent gas, observing that the electrode must be conditioned before use through several cycles of humidification–dehumidification.

Huang et al. [125] faced the problem of humidity quantification in the agricultural sector, in which there are many interfering agents. The authors encapsulated rGO under a layer of GO deposited by spray coating, observing a reduction in cross-sensitivity towards both NH_3_ and ethanol and retaining a good response and high sensitivity of up to 0.4% RH. As for the detection of other species, GRM tailoring significantly helps the sensing process, as proven by decoration with metal oxides [70,72], metal nanoparticles or organic fragments [75].

### 3.4. Pristine Graphene and GRM Sensing Performance in Electrochemical Gas Detection: NH_3_ and NO_x_

**Figure 6:**
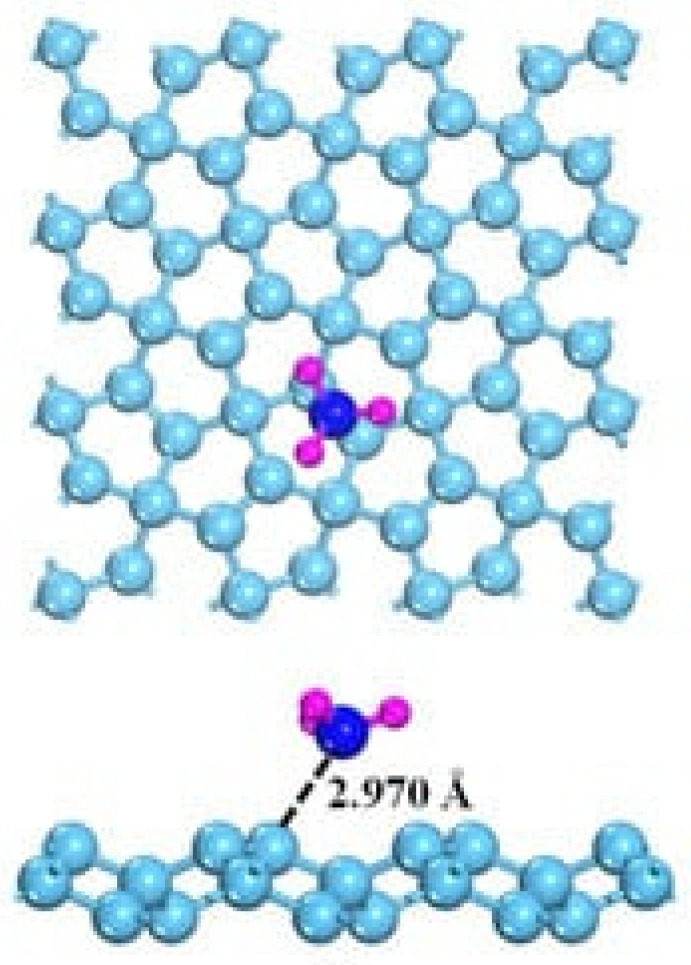
Computational simulation of the interaction between NH_3_ and graphene at 25 °C(carbon atoms were reported as light blue, nitrogen atoms were reported as dark blue and hydrogen ones as purple). Reprinted with all permission from Chen et al. [128].

The detection of NH_3_ is highly interesting due to the harmfulness of this gas [126,127]. The interaction with graphene is also, in this case, the key to understanding how to optimize the NH_3_ sensing process. As shown in Figure 6 [128], NH_3_ interaction with graphene occurs mainly through weak hybridizations between graphene and NH_3_ p orbitals. Accordingly, NH_3_ acts as an electron donor, but pristine graphene is not able to promote both good adsorption and an efficient small transfer charge, resulting in poor detection ability.

The interaction between NH_3_ and graphene was evaluated by Song et al. [76]. The authors investigated the connection between sensitivity and graphene layers using single-layer, double-layer and multilayer graphene and 12,500 ppm of NH_3_. The results showed that the electron transfer is three times higher in single-layer graphene than in the other species due to the easy rearrangement of charge density. Su et al. [77] used a similar approach for the production of a GO layered sensor. Also, in this case, the single-layer material showed the best performance, with a linear range from 5 to 100 ppm and a sensitivity over 15% greater than the multilayered GO. Among GRMs, fluorinated graphene also showed remarkable performance for NH_3_ sensing, showing a 7% change in the resistive response, while pristine GO did not show any sensing ability [78]. This behavior was due to the lower Fermi level of GO and to the increment of hole density in fluorinated GO. Alternatively, Li et al. [79] doped graphene using phosphorous for NH_3_ sensing. The authors reported an increment in performance with a reduction in both response and recovery time up to 71% and 73%, respectively and a detection limit of 69 ppb. Furthermore, the phosphorous-doped system showed a remarkable combination of repeatability, stability, and selectivity. The functionalization using both organic [80] and inorganic [81,82] species is also, in this case, a powerful tool for enhancing the electrochemical properties of GRMs.

NO_x_ species represent the other great family of hazardous nitrogen-based gases [129]. Matatagui et al. [83] faced the issue represented by the detection of NO_2_ in the presence of NH_3_ using a multilayered porous graphene electrode. After photoactivation, the authors reported a change in resistance of 16% in the presence of 0.5 ppm NO_2_, with a detection limit of around 25 ppb. Interestingly, the response to both NH_3_ (50 ppm) and H_2_O (TH 33%) is negligible. The graphene decoration allowed for further improvement in the performance of the sensor by including silicon [84] or phosphorus [85], reaching a response of up to 22% and 59% resistance change using 50 ppm of NO_2_.

Duy et al. [86] deeply explored the tailoring of the rGO surface with metal frameworks, including TiO_2_ nanoparticles and WO_3_, WS_2_, and MoS_2_ nanoflakes using cellulose as a binder. The authors reported a sensitivity boost towards NO_2_, improving the detection limit up to 0.7 ppm using MoS_2_ nanoflakes. ZnO oxide nanoparticles have also been diffusely used for the same scope [130,131,132] with poor results if compared with other nanostructures, such as cobalt supported on rGO [87], chromium-tailored graphene [133], and mixed iron and cobalt oxide [88] or copper [89] onto graphene.

### 3.5. Pristine Graphene and GRM Sensing Performance in Electrochemical Gas Detection: H_2_S and SO_2_

**Figure 7:**
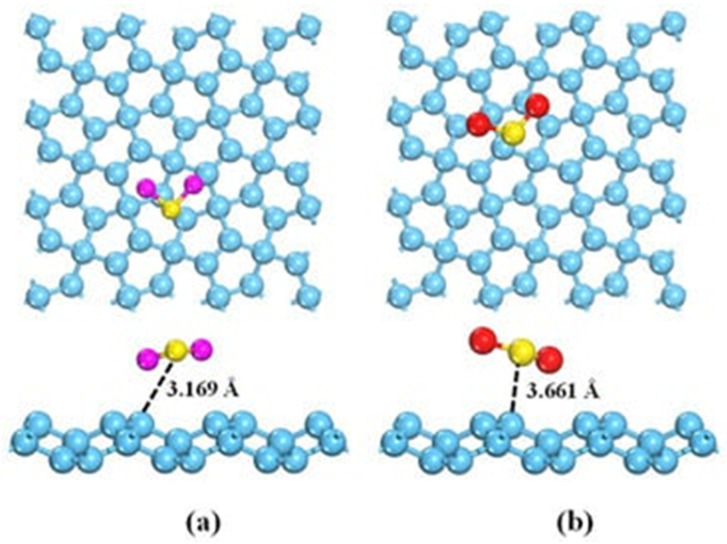
Computational simulation of the interaction between (**a**) H_2_S and (**b**) SO_2_ and graphene at 25 °C(carbon atoms were reported as light blue, sulphur atoms were reported as yellow, oxygen atoms were reported as red and hydrogen ones as purple. Reprinted with all permission from del Chen et al. [128].

As previously described for NH_3_, the interaction between GRMs and H_2_S or SO_2_ occurs mainly by p-orbital interactions with the π graphene system. As shown in Figure 7a,b, H_2_S or SO_2_ interact with different geometry and distances due to the differences in the electron acceptor behavior of SO_2_, while H_2_S acts as an electron donor. Nevertheless, pristine graphene is only poorly able to detect them as a consequence of very weak interactions with them [134].

As reported in the computational research carried out by Liu et al. [87], the insertion of a dopant agent such as aluminum atoms together with Stone–Wales defects can be beneficial for boosting the adsorption of SO_2_, and a similar result was reported for H_2_S [135].

Ugale et al. [90] approached the detection of H_2_S using ZnO-tailored rGO fibers, reaching a detection limit of 8 ppm but a poor selectivity in the presence of NO. Song et al. [91] obtained better performance using SnO_2_ supported on rGO, achieving a 33% response in 2 s using 50 ppm of H_2_S. Furthermore, this system was totally reversible at 22 °C, allowing for long-time use. Similar results were obtained by using Co_3_O_4_-tailored graphene nanospheres [92] or by using copper or WO_3_ supported on rGO [92]. Even if the H_2_S and SO_2_ sensing using GRMs is a field of great interest for both health and safety, the majority of the published research is currently focused more on the computational point of view rather than the applicative process, creating a perilous gap in the research [135].

It is noteworthy that Kumar et al. [93,95] deeply investigated the utilization of rGO for the detection of SO_2_, achieving a limit of detection of up to 5 ppm by using annealed rGO.

### 3.6. Future Outlook for Pristine Graphene and GRM Gas Sensors: Wearable Devices

GRM-based gas sensors are still far from being affordable, but they show interesting perspectives for application in the production of wearable devices [136]. Wearable GRM gas sensors combine flexibility and light weight, enabling the creation of sensors integrated into clothing or into accessories and providing a non-intrusive solution for continuous gas monitoring [137,138].

**Figure 8:**
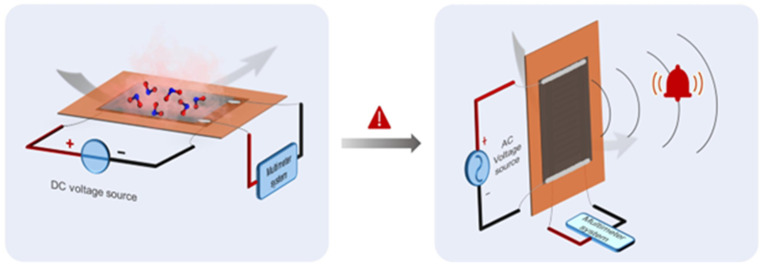
Stretchable laser-induced graphene-based humidity sensor. Reprinted with all permission from del Peng et al. [140].

This sensor family will allow for real-time monitoring, enabling continuous tracking of environmental and personal exposure to gases [139] and promoting healthcare for several activities, including those in which workers can be exposed to hazardous gases. As reported in Figure 8, Peng et al. [140] produced a humidity sensor based on laser-induced graphene that is stretchable and able to operate in real industrial environments.

**Figure 9:**
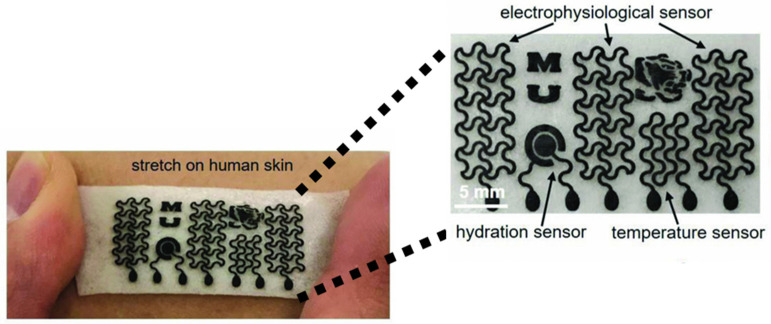
Biocompatible gas sensors based on GRM millimetric circuits. Reprinted with all permission from del Sun et al. [142].

The wearable GRM gas sensor has another key feature, i.e., high energy efficiency, that allows for prolonged usage without the need for frequent recharging [141]. As shown in Figure 9, Sun et al. produced a GRM-based platform able to exploit several functions, such as real-time monitoring of temperature, hydration and sweat due to the remarkable water vapor permeability.

Monitoring using high-performance electrodes will allow a significant improvement in the safety of operation in vulnerable environments such as the semiconductor and food industries, where the atmosphere should be continuously monitored, and for chemical industries operating with complex gas mixtures at high temperatures and pressures.

## 4. Conclusions

The field of gas sensors is of paramount relevance for both health and safety. The development of new high-performance materials is the key to the future of the field, and GRMs can play a relevant and active role. Nowadays, their superior performance is counterbalanced by their high cost, but a great effort has been devoted to making their industrial production more economically feasible. Nevertheless, the usage of GRM-based gas sensors can easily reach all those applications in which performance is more important than economics. Furthermore, the field of wearable gas sensors for monitoring both people’s metabolism and the surrounding environment is a field of application where GRMs represent the state of the art.

We believe in the near-future scenario in which these materials will reach and improve other key sectors of daily life.

## Figures and Tables

**Table 1:** Overview of the key features of graphene and GRMs in electrochemical gas sensing.

Gas	Material	Highlights	References
CO_2_	Chemical vapor-deposited single-layer graphene	▪ Poor responsivity. ▪ Negligible sensitivity in presence of water. ▪ Linear range up 2000 ppm.	[44]
	Exfoliated graphite nanoplatelet	▪ Good sensitivity in presence of water. ▪ Linear range 10–200 ppm.	[45]
	Double-layer graphene	▪ No cross-sensitivity with H_2_O up to 3% relative humidity.	[46]
	GO	▪ Cheap. ▪ Robust. ▪ Linear range 400–4000 ppm.	[47]
	rGO	▪ Response of up to 71% in N_2_. ▪ Response of up to 15% in air.	[48]
	Inorganics (zinc, titanium, molybdenum)-tailored graphene	▪ Operating from 10 to 60 °C. ▪ Operating up to 97% of relative humidity. ▪ Linear range 300–1100 ppm.	[49]
CO	Palladium-tailored rGO	▪ Operating at 150 °C. ▪ Operating up to 71% of relative humidity. ▪ Linear range 200–1100 ppm.	[50]
	Palladium and tin oxide-tailored rGO	▪ Slow response rate of 70 s. ▪ Operating up to 85% of relative humidity due to the formation of surface channels. ▪ Linear range up to 400 ppm.	[51]
	Zinc oxide-tailored rGO	▪ Fast response up to 9 s. ▪ Response of up to 82%. ▪ Recovery time of 14 s. ▪ Linear range 1–1000 ppm.	[52]
	Manganese oxide-tailored rGO	▪ Fast response up to 3 s. ▪ Response up to 70 s. ▪ Linear range 1–1000 ppm.	[53]
	Tin oxide-tailored rGO	▪ Good selectivity over ammonia, hydrogen, and water at 25 °C.	[54]
	Nickel manganate rod-tailored rGO	▪ Ultra-low detection limit (0.6–1 ppm). ▪ Linear range 20–250 ppm.	[55]
	Copper oxide-tailored rGO	▪ Ultra-low detection limit (0.3 ppm). ▪ Slow response rate up to 76 s. ▪ Slow recovery up to 247 s.	[56]
	Cobalt and iron oxide-tailored rGO	▪ Fast response up to 0.5 s. ▪ Linear range 10–40,000 ppm.	[57]
	rGO	▪ Response up to 71% with 30 ppm of CO. ▪ Sensitivity 10 ppm. ▪ Recovery time of up to 30 s.	[58]
	Poly(3,4-ethylenedioxythiophene)-tailored GO	▪ Recovery of up to 42 s. ▪ Linear range 20–270 ppm.	[59,60]
	Poly(N-methyl pyrrole)-tailored rGO	▪ Recovery of up to 36 s. ▪ Detection limit of 1 ppm. ▪ Linear range 10–275 ppm.	[61]
	Metal organic framework-tailored rGO	▪ Fast response up to 30 s. ▪ Fast recovery up to 70 s. ▪ Great durability in CO atmosphere for over 30 days. ▪ Sensitivity of 25 ppm.	[62]
H_2_	Palladium nanoparticles onto single-layer graphene	▪ Response of 33% in 1000 ppm of at H_2_ 25 °C. ▪ Sensitivity of 20 ppm.	[63]
	Palladium nanoparticles onto 3D-GRMs	▪ Response of 41.9% under 3% H_2_ at 25 °C. ▪ Easy to produce.	[64]
	Platinum nanoparticle-decorated rGO	▪ Response of 8% under 0.5% H_2_ at 50 °C. ▪ Fast recovery up to 104 s.	[65]
	Platinum nanoparticles onto 3D-GRMs	▪ High sensitivity. ▪ Fast response up to 9 s. ▪ Fast recovery up to 10 s. ▪ Good linearity in the range from 1 to 100 ppm.	[66]
	Tin oxide onto platinum nanoparticle-decorated rGO	▪ Enhanced response compared with palladium-decorated rGO. ▪ Enhanced sensitivity compared with palladium-decorated rGO.	[67]
	Tungsten-decorated GO	▪ Response of 50 mV in presence of H_2_ (100 ppm). ▪ Use in air atmosphere. ▪ Detection limit of 11 ppm.	[68]
	Zinc oxide-decorated GO	▪ Fast response up to 114 s. ▪ Short recovery time up to 30 s. ▪ Detection limit of 4 ppm.	[69]
H_2_O	Vertically aligned graphene arrays	▪ Sensitivity related to relative humidity. ▪ Improved performance for relative humidity over 70%.	[70]
	GO	▪ Fastest response reported of up to 0.18 s. ▪ Recovery time of 0.3 s. ▪ Operativity from 37% up to 98% relative humidity.	[71]
	Zinc oxide-tailored graphene foam	▪ High stability. ▪ High regenerability. ▪ Linearity from 20% up to 95% relative humidity.	[72]
	Zinc oxide-tailored GO	▪ Response of up to 1 s. ▪ Linearity from 20% up to 95% relative humidity.	[73]
	Silver nanoparticle-tailored GO	▪ Sensitivity of 26 nF/% RH. ▪ Linear range from 11% up to 87% relative humidity.	[74]
	N-[4-morpholinecarboximidamidoyl] carboximidamidoylated GO	▪ Response of up 20. ▪ Recovery time of 2 s.	[75]
NH_3_	Chemical vapor-deposited graphene	▪ Properties related to graphene layer numbers.	[76]
	GO	▪ Properties related to graphene layer numbers. ▪ Linear range from 10 to 100 ppm. ▪ Single-layer performed better than double- and multilayer electrodes.	[77]
	Fluorinated GO	▪ Improvement over 7% performance compared with GO.	[78]
	Phosphorous-doped graphene	▪ Limit of detection of 69 ppb. ▪ Improvement over 70% of electrochemical performance compared with pristine graphene.	[79]
	Aniline-tailored graphene	▪ Easy fabrication. ▪ Response of 37% in 50 ppm of NH_3_.	[80]
	Zinc oxide on rGO	▪ Fast response up to 2 s. ▪ Fast recovery up to 13 s in 350 ppm of NH_3_. ▪ Detection limit of 10 ppm.	[81]
	CuFe_2_O_4_-tailored rGO	▪ Fast response up to 3 s. ▪ Fast recovery up to 6 s. ▪ High selectivity (over 5 times) for NH_3_ in presence of CH_3_OH, CO_2_, benzene. ▪ Limit of detection of 5 ppm.	[82]
NO_2_	Multilayered porous graphene	▪ Selective for NO_2_ in presence of NH_3_. ▪ Detection limit of 25 ppb. ▪ Response independent from relative humidity.	[83]
	Silicon-doped graphene	▪ High response value of up to 22 in 50 ppm of NO_2_. ▪ Fast response up to 126 s. ▪ Fast recovery up to 378 s. ▪ Linear range from 18 ppb up to 300 ppm. ▪ Good selectivity.	[84]
	Phosphorous-doped graphene	▪ High response value of up to 59% in 50 ppm of NO_2_. ▪ Detection limit 1 ppm.	[85]
	Metal frameworks on rGO	▪ Detection limit 0.7 ppm. ▪ Selective for NO_2_ in presence of NH_3_. ▪ Nonselective for NO_2_ in presence of NO.	[86]
	Cobalt hydroxide-tailored rGO	▪ High sensitivity of 70% exposed to 100 ppm of NO_2_. ▪ Detection limit of 1 ppm.	[87]
	Mixed iron and cobalt oxide-tailored graphene	▪ Good response of up to 50 s. ▪ Detection limit of 1 ppm.	[88]
	Copper nanoparticle-tailored graphene	▪ Great reproducibility. ▪ Detection limit of 30 ppb. ▪ Slow response.	[89]
H_2_S	Zinc oxide onto rGO	▪ Poor selectivity in presence of NO. ▪ Detection limit of 8 ppm.	[90]
	Tin oxide onto rGO	▪ Fast response in 2 s using 50 ppm of H_2_S. ▪ Response up to 30%. ▪ Regenerable.	[91]
	Cobaltite supported on graphene nanospheres	▪ Response of 30% in presence of 50 ppm of H_2_S. ▪ Linear range from 1 to 70 ppm.	[92]
SO_2_	Annealed rGO	▪ Stability over 30 days in sulphur dioxide atmosphere. ▪ Detection limit 5 ppm.	[93]
	Sheets of GO	▪ Moderate response up to 65 s. ▪ Fast recovery up to 100 s. ▪ Detection limit of up to 15 ppm.	[94]
	rGO	▪ Response of up to 47% in 50 ppm of SO_2_. ▪ Detection limit of up to 5 ppm.	[95]
